# Canadian Internal Medicine Ultrasound (CIMUS) consensus statement: recommendations for mandatory ultrasound competencies for ultrasound-guided thoracentesis, paracentesis, and central venous catheterization

**DOI:** 10.1186/s13089-024-00363-8

**Published:** 2024-03-22

**Authors:** Canadian Internal Medicine Ultrasound, Michael H. Walsh, Marko Balan, Steven J. Montague, Dayna Butler, Barry Chan, Allen Tran, Julien Viau-Lapointe, Jeffrey Wiseman, Hugh Traquair, Jeffrey Yu, Pierre-Alexis Lépine, Janeve Desy, Tyler B. Friesen, Mathilde Gaudreau-Simard, Linden Kolbenson, Dev Jayaraman, Sonja Lubbers, Michael Mayette, Michael Sattin, Leo M. Smyth, Monty Sandhu, Gillian Spiegle, Audrey Lacasse, Ada W. Lam, Katie Wiskar, Shane Arishenkoff, Jonathan Wong, Irene W. Y. Ma

**Affiliations:** Division of General Internal Medicine, University of Calgary Cumming School of Medicine, Calgary, AB Canada

## Abstract

**Objectives:**

To develop a Canadian Internal Medicine Ultrasound (CIMUS) consensus statement on recommended mandatory point-of-care ultrasound (POCUS) competencies for ultrasound-guided thoracentesis, paracentesis, and central venous catheterizations (CVC) for internal medicine physicians.

**Methods:**

The 2022 CIMUS group consists of 27 voting members, with representations from all 17 Canadian academic institutions across 8 provinces. Members voted in 3 rounds on 46 procedural competencies as “mandatory, must include”, “optional, could include” or “superfluous, do not include”. These 46 competencies included 6 general competencies that apply to all POCUS-guided procedures, 11 competencies for thoracentesis, 10 competencies for paracentesis, and 19 competencies for CVC.

**Results:**

In the first round, members reached consensus on 27 competencies (5 general, 6 thoracentesis, 8 paracentesis, 8 CVC). In the second round, 10 competencies (1 general, 2 thoracentesis, 1 paracentesis, 6 CVC) reached consensus. In the third round, 2 additional competencies (1 paracentesis, 1 CVC) reached consensus for being mandatory and 3 as optional (1 thoracentesis and 2 CVC). Overall, a total of 28 competencies reached consensus as mandatory, 3 as optional, while 11 competencies reached consensus as superfluous. Four competencies did not reach consensus for either inclusion or exclusion.

**Conclusions:**

The CIMUS group recommends 28 competencies be considered mandatory and 3 as optional for internal medicine physicians performing POCUS guided thoracentesis, paracentesis, and CVC placement. National curriculum development and implementation efforts should include training these mandatory competencies.

## Introduction

Thoracentesis, paracentesis, and central venous catheterization (CVC) are bedside procedures commonly performed by internal medicine physicians and residents in training. In Canada, the standards for specialists in internal medicine to safely perform these procedures is set by the Royal College of Physicians and Surgeons of Canada [1]. The use of ultrasound-guidance for these procedures is now considered the standard of care and recommended by multiple societies and organizations, given its association with higher success and lower complication rates [2–10].

Despite ultrasound guidance being considered the standard of care, *how* specifically to use point-of-care ultrasound (POCUS) and *what* the associated mandatory competencies are have not been clearly defined [1]. POCUS competencies necessary for the performance of bedside procedures range from simple indirect guidance techniques to more advanced ones, such as assessment for vessels to avoid prior to needle puncture and direct ultrasound guidance through real-time visualization of the needle tip, guidewire, and catheter [11–13]. The lack of clarity in expected mandatory competencies required for the safe performance of bedside procedures may result in variable training standards across internal medicine residency programs in Canada. Therefore, the goal of this consensus statement is to determine mandatory POCUS competencies for the performance of ultrasound-guided thoracentesis, paracentesis, and CVC for Canadian internal medicine specialists to guide curriculum development for residency training programs.

## Methods

The Canadian Internal Medicine Ultrasound group (CIMUS) members who participated in this initiative are individuals identified in March 2022 by their internal medicine division leads as having a POCUS leadership role at their institution. All 17 post-graduate academic institutions across Canada were invited to partipate. Participants were POCUS experts, educational experts, and/or senior level trainees with POCUS leadership roles. Members disclosed relevant conflict of interests and only one member (KW) had relevant conflicts to declare.

Participants met in person or virtually in a 4-h consensus meeting during the Canadian Society of Internal Medicine Annual Meeting in Victoria, B.C., Canada, on October 13, 2022. The objectives of the meeting were to establish consensus-based recommendations on mandatory POCUS competencies for ultrasound-guided thoracentesis, paracentesis, and CVC for Canadian internal medicine training programs. The consensus process was facilitated by an academic general internal medicine specialist with POCUS, medical education, and consensus methods expertise (IM). The facilitator did not participate in the voting process.

Prior to the meeting, relevant POCUS competencies were selected from a previously published list of comprehensive competencies relating to thoracentesis, paracentesis, and CVC [11]. Competencies were then augmented by a literature review of key relevant recommendations available since the initial publication [3, 5, 7]. From this list of candidate competencies, participants were asked to vote if each competency should be “mandatory, must include,” “optional, could include,” or “superfluous, do not include.” Abstentions were permitted. Participants were asked to prioritize competencies that are deemed necessary for patient safety and procedural success, as well as consider *feasibility for teaching* within the Canadian internal medicine educational context and *learnability* for our Canadian postgraduate medical trainees. Feasibility considerations included those such as the availability of time in the curriculum, faculty, equipment, and educational resources. Learnability considerations included those such as the baseline level of learner knowledge and skills and the complexity and skill level required to perform the competency in question. Voting was done online (Qualtrics, Provo, UT). We determined a priori to conduct no more than three rounds of voting and defined consensus as 70% of agreement or higher amongst members [14]. Competencies that did not reach consensus during Round 1 were included in subsequent rounds of voting. When competencies were mutually exclusive, competencies that were no longer relevant were removed. Quantitative and anonymized qualitative results were presented to the group in Rounds 2 and 3.

## Results

A total of 27 voting members, with representation from all 17 academic institutions across Canada participated in the meeting. Demographics of the 27 members are presented in Table 1.

**Table 1:** Baseline characteristics of the 27 voting members who participated in the study

Characteristics	N (%)
Gender	
Male	18 (67)
Female	9 (33)
Duration of POCUS fellowship training	
None	16 (59)
1–6 months	6 (22)
> 6 months	5 (19)
Duration of POCUS experience	
Not using POCUS	1 (4)
1–2 years	4 (15)
3–5 years	7 (26)
6–10 years	15 (56)
Postgraduate degree training	
Medical Education	6 (22)
Public Health/Epidemiology	1 (4)
Health Administration	1 (4)
Subspecialty Training (in addition to internal medicine / general internal medicine)	
Critical care	5 (19)
Pulmonary	1 (4)
Obstetric medicine	1 (4)

### Round 1

In Round 1, 26 of the 27 members (96%) voted. A total of 46 competencies were considered: 6 general competencies that apply to all three procedures, 11 for thoracentesis, 10 for paracentesis, and 19 for CVC (Table 2).

**Table 2:** Round 1 results

Competency	Mandatory, no. (%)	Optional, no. (%)	Superfluous, no. (%)
General competencies			
Know which probe(s) to use and probe orientation	25/26 (96)*	1/26 (4)	0
Basic knobology (depth/gain)	26/26 (100)*	0	0
Appropriate infection prevention and control measures	18/26 (69)	5/26 (19)	2/26 (8)
Appropriate sheathing of transducers for real-time guidance	20/25 (80)*	2/25 (8)	3/25 (12)
Recognizes limitations (e.g. when procedure cannot be performed or should be performed by a more experienced proceduralist)	25/26 (96)*	1/26 (4)	0
Able to correlate findings on ultrasound with surface anatomy	19/26 (73)*	5/26 (19)	2/26 (8)
Thoracentesis			
Can identify the spine sign (e.g. vs. mimickers and false positives of pleural effusion such as drop out artifact)	19/26 (73)*	7/26 (27)	0
Characterization of pleural effusion (septations/etc.)	18/26 (69)	8/26 (31)	0
Assessment to include probe sliding/two planes	20/25 (80)*	3/25 (12)	2/25 (8)
Assessment to include deep breaths (lung movement) and diaphragm movement	14/26 (54)	10/26 (38)	2/26 (8)
Can identify location of diaphragm/intra-abdominal organs/lung tip	25/26 (96)*	1/26 (4)	0
Ruling out vasculature (e.g. intercostal vessels)	11/26 (42)	14/26 (54)	1/26 (4)
Indirect guidance	25/26 (96)*	1/26 (4)	0
Direct real-time guidance^†^	1/26 (4)	8/26 (31)	17/26 (65)
Assessing for pneumothorax pre- and post-	7/26 (27)	12/26 (46)	7/26 (27)
Sinusoidal sign	0	5/25 (20)	20/25 (80)*
Pleural effusion size estimation	1/25 (4)	12/25 (48)	12/25 (48)
Paracentesis			
Can identify abdominal free fluid	25/26 (96)*	1/26 (4)	0
Assessment to include probe sliding/two planes	18/26 (69)	7/26 (26)	1/26 (4)
Assessing for location of structures to avoid (liver, spleen, bladder, kidney, bowel)	24/26 (92)*	2/26 (8)	0
Assessment to include compression to ensure depth of fluid collection remains sufficient	17/26 (65)	8/26 (31)	1/26 (4)
Ruling out vasculature (e.g. inferior epigastrics/collaterals)	20/26 (77)*	6/26 (23)	0
Indirect guidance	26/26 (100)*	0	0
Real time guidance (either in-plane or out-of-plane)^†^	1/26 (4)	8/26 (31)	17/26 (65)
Real-time in-plane guidance	0	4/25 (16)	21/25 (84)*
Real-time out-of-plane guidance	0	4/25 (16)	21/25 (84)*
Recognizes mimickers of free fluid (e.g. intraluminal fluid, perinephric fat)	22/25 (88%)*	3/25 (12)	0
Central venous catheterization (CVC)			
Can differentiate between artery vs. vein	26/26 (100)*	0	0
Assessment to include ruling out deep vein thrombosis (in the access site vein, not thrombosis in the legs)	15/26 (58)	10/26 (38)	1/26 (4)
Identify appropriate insertion site based on anatomy (not just where vein is largest, but if location is too low and at high risk of pneumothorax)	22/26 (85)*	4/26 (15)	0
Can identify underlying lung (for internal jugular and subclavian sites)	20/26 (77)*	5/26 (19)	1/26 (4)
Creep technique for all real-time guidance maneuvers	17/25 (68)	7/25 (28)	1/25 (4)
Real time guidance for internal jugular CVC	24/26 (92)*	2/26 (8)	0
Internal jugular CVC: either in-plane or out-of-plane	17/25 (68)	7/25 (28)	1/25 (4)
Internal jugular CVC: in-plane	2/24 (8)	14/24 (58)	8/24 (33)
Internal jugular CVC: out-of-plane	7/24 (29)	8/24 (33)	9/24 (34)
Subclavian CVC: real-time guidance	10/25 (40)	9/25 (36)	6/25 (24)
Subclavian CVC: indirect guidance	4/25 (16)	12/25 (48)	9/25 (36)
Femoral CVC: real-time guidance	22/26 (85)*	4/26 (15)	0
Femoral CVC: indirect guidance^†^	7/24 (29)	8/24 (33)	9/24 (38)
Assessment to include confirming location of wire intra-vein with ultrasound	25/26 (96)*	1/26 (4)	0
Assessment to include confirming catheter is intra-vein with ultrasound	10/26 (38)	11/26 (42)	5/26 (19)
Assessing for pneumothorax pre- and post-	6/25 (24)	11/25 (44)	5/25 (2)
Saline flush test	3/26 (12)	10/26 (38)	13/26 (50)
Assessing for direction of catheter towards superior vena cava	2/26 (8)	7/26 (27)	17/26 (65)
Performs bicaval view to visualize the catheter tip	1/25 (4)	4/25 (16)	20/25 (80)*

^*^Consensus achieved

^†^Removed after Round 1 because of its relation to another competency that reached consensus

Consensus for inclusion was achieved for 5 of the 6 general competencies (Table 2). For thoracentesis, consensus was achieved for 6 of the 11 competencies. Four competencies reached consensus to be included. As performing indirect guidance was agreed upon as mandatory, direct real-time ultrasound guidance was removed as a mandatory competency. For paracentesis, consensus was achieved for 8 of the 10 competencies. For CVC, consensus was achieved for 8 of 19 competencies. Of these, 6 reached consensus for inclusion. Given real-time guidance was deemed mandatory for internal jugular vein and femoral vein catheterizations, indirect guidance was removed as a mandatory competency. Consensus was also achieved for excluding the performance of the bicaval view to visualize the catheter tip as a mandatory competency.

### Round 2

All 27 members voted in Round 2. The single item considered within general competency achieved consensus for inclusion (Table 3). For thoracentesis, consensus for inclusion was achieved on 2 of the 5 competencies. For paracentesis, 1 of the 2 competencies achieved consensus to be included after wording revision: assessment to include probe sliding and scanning in two planes to ensure a sufficient area surveyed. For CVC, consensus was achieved for 6 of the 11 competencies, with 2 competencies meeting consensus for inclusion after wording revisions: creep technique for real-time guidance (i.e. maintaining visualization of the needle tip all the way into the vein) and that the internal jugular vein can be done either in- or out-of-plane. As such, two additional competencies (i.e. internal jugular vein must be done only in-plane and only out-of-plane) were removed. Two competencies met consensus for exclusion.

**Table 3:** Round 2 results

Competency	Mandatory, no. (%)	Optional, no. (%)	Superfluous, no. (%)
General competencies			
Appropriate infection prevention and control measures	26/27 (96)*	1/27 (4)	0
Thoracentesis			
Characterization of pleural effusion (septations/etc.)	21/27 (78)*	5/27 (19)	1/27 (4)
Assessment to include deep breaths (lung movement) and diaphragm movement	19/27 (70)*	6/27 (22)	2/27 (7)
Ruling out vasculature (e.g. intercostal vessels)	7/27 (26)	17/27 (63)	3/27 (11)
Assessing for pneumothorax pre- and post-	4/27 (15)	18/27 (67)	5/27 (19)
Pleural effusion size estimation	0	11/27 (41)	16/27 (59)
Paracentesis			
Assessment to include sliding/two planes *to ensure a sufficient area surveyed*	22/26 (85)*	3/26 (11)	1/26 (4)
Assessment to include compression to ensure depth of fluid collection remains sufficient	17/27 (63)	8/27 (30)	2/27 (7)
Central venous catheterization (CVC)			
Assessment to include ruling out deep vein thrombosis (in the access site vein, not thrombosis in the legs)	16/27 (59)	11/27 (41)	0
Creep technique (to ensure visualization of needle all the way in) for all real-time guidance maneuvers	23/27 (85)*	4/27 (15)	0
Internal jugular CVC: either in-plane or out-of-plane	23/27 (85)*	3/27 (11)	1/27 (4)
Internal jugular CVC: in-plane^†^	0	9/26 (35)	17/26 (65)
Internal jugular CVC: out-of-plane^†^	5/27 (19)	6/27 (22)	16/27 (59)
Subclavian CVC: real-time guidance	5/24 (21)	13/24 (54)	6/24 (25)
Subclavian CVC: indirect guidance	2/24 (8)	13/24 (54)	9/24 (38)
Assessment to include confirming catheter is intra-vein with ultrasound	8/27 (30)	14/27 (52)	5/27 (19)
Assessing for pneumothorax pre- and post-	3/27 (11)	16/27 (59)	8/27 (30)
Saline flush test	0	6/27 (22)	21/27 (78)*
Assessing for direction of catheter towards superior vena cava	0	4/27 (15)	23/27 (85)*

^*^Consensus achieved

^†^Removed after Round 2 because of its relation to another competency that reached consensus

### Round 3

In the final round, all 27 participants voted. For the 3 items remaining for thoracentesis (Table 4), assessing for pneumothorax pre- and post-procedure reached consensus as being “optional.” The other 2 items did not reach consensus. The single item considered for paracentesis met consensus for inclusion. Of the 5 items remaining for CVC, 1 met consensus for inclusion, and 2 reached consensus as “optional.” The other 2 items did not reach consensus. Final items reaching consensus are presented in Table 5.

**Table 4:** Round 3 results

Competency	Mandatory, no. (%)	Optional, no. (%)	Superfluous, no. (%)
Thoracentesis			
Ruling out vasculature (e.g. intercostal vessels)	8/27 (30)	18/27 (67)	1/27(4)
Assessing for pneumothorax pre- and post-	3/27 (11)	20/27 (74)*	4/27 (15)
Pleural effusion size estimation	1/26 (4)	8/26 (31)	17/26 (65)
Paracentesis			
Assessment to include compression to ensure depth of fluid collection remains sufficient	21/27 (77)*	5/27 (19)	1/27 (4)
Central venous catheterization (CVC)			
Assessment to include ruling out deep vein thrombosis (in the access site vein, not thrombosis in the legs)	19/27 (70)*	8/27 (30)	0
Subclavian CVC: real-time guidance	6/23 (26)	14/23 (61)	3/23 (13)
Subclavian CVC: indirect guidance	2/23 (9)	16/23 (69.6)	5/23 (22)
Assessment to include confirming catheter is intra-vein with ultrasound	2/27 (7)	23/27 (85)*	2/27 (7)
Assessing for pneumothorax pre- and post-	4/27 (15)	19/27 (70)*	4/27 (15)

^*^Consensus achieved

**Table 5:** Final recommendation for point-of-care ultrasound competencies for thoracentesis, paracentesis, and central venous catheterization (CVC)

Mandatory general competencies
Know which probe(s) to use and probe orientation
Basic knobology (depth/gain)
Appropriate infection prevention and control measures
Appropriate sheathing of transducers for real-time guidance
Recognizes limitations (e.g. when procedure cannot be performed or should be performed by a more experienced proceduralist)
Able to correlate findings on ultrasound with surface anatomy
Mandatory thoracentesis competencies
Can identify the spine sign (e.g. vs. mimickers and false positives of pleural effusion such as drop out artifact)
Characterization of pleural effusion (septations/etc.)
Assessment to include probe sliding/two planes
Assessment to include deep breaths (lung movement) and diaphragm movement
Can identify location of diaphragm/intra-abdominal organs/lung tip
Indirect guidance
Mandatory paracentesis
Can identify abdominal free fluid
Assessment to include probe sliding/two planes to ensure a sufficient area surveyed
Assessing for location of structures to avoid (liver, spleen, bladder, kidney, bowel)
Assessment to include compression to ensure depth of fluid collection remains sufficient
Ruling out vasculature (e.g. inferior epigastrics/collaterals)
Indirect guidance
Recognizes mimickers of free fluid (e.g. intraluminal fluid, perinephric fat)
Mandatory central venous catheterization (CVC)
Can differentiate between artery vs. vein
Assessment to include ruling out deep vein thrombosis (in the access site vein, not thrombosis in the legs)
Identify appropriate insertion site based on anatomy (not just where vein is largest, but if location is too low and at high risk of pneumothorax)
Can identify underlying lung (for internal jugular and subclavian sites)
Creep technique (to ensure visualization of needle all the way in) for all real-time guidance maneuvers
Real time guidance for internal jugular CVC
Internal jugular CVC: either in-plane or out-of-plane
Femoral CVC: real-time guidance
Assessment to include confirming location of wire intra-vein with ultrasound
Optional thoracentesis competency
Assessing for pneumothorax pre- and post-
Optional CVC competencies
Assessment to include confirming catheter is intra-vein with ultrasound
Assessing for pneumothorax pre- and post-

## Discussion

In this consensus study, 27 Canadian internal medicine POCUS and education leaders across 17 institutions considered a total of 46 POCUS-related competencies for three commonly performed bedside procedures in internal medicine (thoracentesis, paracentesis, and CVC). Of these, participants reached consensus on 28 competencies as mandatory, 3 as optional, and 11 as superfluous or were removed. Four competencies did not reach consensus. Given CIMUS has representation from all 17 training programs across Canada, these agreed upon competencies could be considered foundational in guiding national training curriculum development efforts.

Our group aimed to identify the *minimum* number of competencies that should be considered mandatory for residents who are often POCUS novices. These recommendations should not be used to limit programs from teaching additional competencies. Indeed, a number of items that did not reach consensus might be important competencies to consider. For example, ruling out vessels prior to performing a thoracentesis did not reach consensus in our panel as mandatory. In one study, evaluation for intercostal vessels with Doppler imaging led to an alteration of needle insertion site in over 15% of cases [15]. Given that the competencies required for vessel assessment are similar for both paracentesis and thoracentesis, and that vessel assessment reached consensus for paracentesis, it should thus require minimal additional training efforts from educators to teach vessel assessment for thoracentesis. Nonetheless, our panel’s decision is concordant with current practice, where the use of Doppler to identify intercostal vessels is not considered mandatory in current position statements [3, 16]. Despite the lack of consensus in our group and others, it may still be prudent for individual practitioners with this competency to continue to rule out vasculature prior to needle insertion for thoracentesis. Similarly, assessing for pneumothorax pre- and post-procedure did not reach consensus for CVC or thoracentesis. The reasons for this lack of consensus are not clear. It is possible that the skills required to definitively diagnose pneumothorax were considered too advanced for our learners, as learners would need to search for and appropriately identify a lung point [17]. Comprehensive pneumothorax assessment would also involve understanding the impact of pre-existing lung findings and patient positioning on diagnostic accuracy [18, 19]. However, ruling out large and clinically significant pneumothorax would be far simpler and relatively easy to teach [20]. Lastly, consensus was reached in Round 1 to consider the sinusoidal sign (the dynamic respiratory movement of the lung within the effusion) as being superfluous. Despite this sign being strongly recommended for the diagnosis of pleural effusion in the International recommendations [19], it is possible that this sign may not be as commonly known in clinical practice as the spine sign [21].

Several limitations to our study must be highlighted. First, our recommendations are expert opinion based and intended to be practical and directly applicable to the current internal medicine training environment in Canada. While a literature search was used to identify competencies to be considered, a systematic review was not conducted and recommendations were not graded. We relied on our participants’ diverse expertise in medical education, POCUS, and leadership skills in deriving our final recommendations. Secondly, our group consists entirely of internal medicine specialists practicing in academic institutions in Canada which may limit the generalizability of our recommendations. Third, participants only received 3 options for each competency: “mandatory, must include,” “optional, could include,” or “superfluous, do not include.” We did not ask our experts to rate competencies on a Likert scale. Finally, our recommendations do not encompass curriculum design and implementation strategies, which would be logical next steps in this work.

## Conclusions

The CIMUS group recommends 28 mandatory POCUS competencies to be included, 3 optional, and 11 competencies to be excluded for three commonly performed bedside procedures in internal medicine (thoracentesis, paracentesis, and CVC). National curriculum development and implementation efforts should take these recommendations into consideration.

## Data Availability

The datasets used and/or analyzed during the current study are available from the corresponding author on reasonable requests.

## Abbreviations

CIMUS: Canadian Internal Medicine Ultrasound Society
CVC: Central venous catheterization
POCUS: Point-of-care ultrasound
